# Strategies for identification of somatic variants using the Ion Torrent deep targeted sequencing platform

**DOI:** 10.1186/s12859-017-1991-3

**Published:** 2018-01-04

**Authors:** Aditya Deshpande, Wenhua Lang, Tina McDowell, Smruthy Sivakumar, Jiexin Zhang, Jing Wang, F. Anthony San Lucas, Jerry Fowler, Humam Kadara, Paul Scheet

**Affiliations:** 1 Departments of Epidemiology, University of Texas MD Anderson Cancer Center, Houston, TX USA; 2 University of Texas School of Public Health, Houston, TX USA; 3 Translational Molecular Pathology, University of Texas MD Anderson Cancer Center, Houston, TX USA; 4 Bioinformatics and Computational Biology, University of Texas MD Anderson Cancer Center, Houston, TX USA; 5 University of Texas Graduate School of Biomedical Sciences, Houston, TX USA; 6 Department of Biochemistry and Molecular Genetics, Faculty of Medicine, American University of Beirut, Beirut, Lebanon

**Keywords:** Next-generation sequencing, Ion torrent, Variant calling strategies, Ion Reporter, Varscan2, MuTect

## Abstract

**Background:**

‘Next-generation’ (NGS) sequencing has wide application in medical genetics, including the detection of somatic variation in cancer. The Ion Torrent-based (IONT) platform is among NGS technologies employed in clinical, research and diagnostic settings. However, identifying mutations from IONT deep sequencing with high confidence has remained a challenge. We compared various computational variant-calling methods to derive a variant identification pipeline that may improve the molecular diagnostic and research utility of IONT.

**Results:**

Using IONT, we surveyed variants from the 409-gene Comprehensive Cancer Panel in whole-section tumors, intra-tumoral biopsies and matched normal samples obtained from frozen tissues and blood from four early-stage non-small cell lung cancer (NSCLC) patients. We used MuTect, Varscan2, IONT’s proprietary Ion Reporter, and a simple subtraction we called “Poor Man’s Caller.” Together these produced calls at 637 loci across all samples. Visual validation of 434 called variants was performed, and performance of the methods assessed individually and in combination. Of the subset of inspected putative variant calls (*n*=223) in genomic regions that were not intronic or intergenic, 68 variants (30%) were deemed valid after visual inspection. Among the individual methods, the Ion Reporter method offered perhaps the most reasonable tradeoffs. Ion Reporter captured 83% of all discovered variants; 50% of its variants were visually validated. Aggregating results from multiple packages offered varied improvements in performance.

**Conclusions:**

Overall, Ion Reporter offered the most attractive performance among the individual callers. This study suggests combined strategies to maximize sensitivity and positive predictive value in variant calling using IONT deep sequencing.

**Electronic supplementary material:**

The online version of this article (doi:10.1186/s12859-017-1991-3) contains supplementary material, which is available to authorized users.

## Background

“Next-generation” sequencing technology (NGS) has facilitated unprecedented discoveries of genomic variation relating to the molecular biology of complex diseases such as cancer [1, 2]. Sequencing of DNA to survey genomic aberrations, including point mutations and copy number alterations, in various malignancies has implicated variants in canonical oncogenes and tumor suppressor genes as drivers [2–4]. Of note, NGS-derived knowledge has underlined therapeutically pliable and actionable alterations in cancer including activating mutations in *EGFR*, *MET*, *BRAF*, *FGFR2* and *FGFR3*, and *PIK3CA* [5–11]. NGS studies highlight the value of incorporating sequencing technologies in personalized treatment strategies and potentially in clinical decision making [12].

The heterologous pathological makeup of clinical specimens represents a unique challenge for surveying genomic aberrations in the clinic [13]. For example, formalin-fixed paraffin embedded (FFPE) cancer specimens, which are typically used for sequencing assays in the clinic, are likely to comprise an admixture of tumor and normal cells. This effectively reduces the allele frequency of the tumor-associated variant to be discerned [14]. In addition to histopathological considerations such as tumor microdissection, it has been suggested that NGS should reach a sufficient sequencing depth, sometimes referred to as “clinical depth,” in order to discern somatic aberrations that exist only in small fractions of the specimen cells. These measures help attain the desired clinical value and goals of NGS technologies [15, 16].

Various NGS platforms have provided methods for deep sequencing [16–18]. One of these platforms, the Ion Torrent (IONT), has been commonly used for deep sequencing in the molecular pathology laboratory setting to survey cancer hot spots or targeted exons of cancer genes (e.g. 409-gene comprehensive cancer panel) [19–22]. However, this platform carries significant sequencing problems. Overall, the quality of base calling accuracy generated by IONT sequencing (quantified as a Phred score) is lower in comparison to other sequencing platforms. Additionally, IONT is more prone to misreading the length of homopolymers compared to other platforms (e.g. Illumina) [23]. These observations warrant the need for better analytical solutions to detect true somatic mutations in IONT sequencing data with high confidence, avoiding false positives that may arise due to sequencing error or inaccurate calling measures. However, methods for high-confidence identification of true mutations from Ion Torrent-based deep sequencing have remained underdeveloped, poorly described, or underutilized [23].

We surmised that development of a somatic variant calling pipeline would offer opportunities for advancing clinical translation of such sequencing platforms. To this end, in this study we compared and contrasted various computational algorithms for calling somatic point mutations (single-nucleotide variants, or SNVs) in tumors, from data generated on the Ion Torrent platform. Using these results, we have derived a working pipeline for identification of high-confidence variants.

## Methods

### Sample description

We surveyed variants in surgically resected non-small cell lung cancer (NSCLC) tumors as well as in normal samples (either uninvolved nasal tissue or blood cells) from four patients with early-stage NSCLC. In addition to the whole tumor sections, six to eight multi-regional intratumoral core needle biopsies (CNBs) from each NSCLC tumor core were also examined. The malignant and normal samples were acquired from early-stage (stages I-III) NSCLC patients evaluated at The University of Texas MD Anderson Cancer Center following informed consent under an institutional review board (IRB) approved protocol. Tumors were classified using the 2004 World Health Organization (WHO) classification system as described previously [24]. All samples were obtained snap-frozen. Table 1 below summarizes the numbers and types of malignant and putatively normal samples (*n*=35). Following identification of somatic variants (see below), one of the CNB specimens in case NSCLC 4 was found to contribute almost half of the total variants in the entire sample set and, thus, was excluded from all downstream analyses.

**Table 1:** Sample description

	Tumor section	Tumor CNB	Normal
NSCLC 1	1	6	Blood
NSCLC 2	1	8	Nasal
NSCLC 3	1	7	Blood
NSCLC 4	1	6	Blood

### Sequencing platform

Genomic DNA was isolated from all samples using the All Prep DNA/RNA kit from Qiagen according to the manufacturer’s instructions. We used 40 ng of DNA from each sample for deep targeted sequencing. Deep sequencing of all exons in a panel of 409 genes [Ion Ampliseq Comprehensive Cancer Panel (CCP, Life Technologies)] was performed using the Ion Torrent Proton sequencing platform from Life Technologies. The CCP comprises over 50% of the Wellcome Trust Sanger Institute Cancer Gene Census: tumor suppressor genes, oncogenes as well as DNA repair genes implicated as potential drivers and targets in cancer based on published sequencing studies of various malignancies [21, 22]. Barcoded libraries (covering 16,000 amplicons distributed among four pools) were generated from 40 ng DNA using the Ion Ampliseq library 2.0 kit according to the manufacturer’s instructions. Libraries were quality controlled using the DNA high sensitivity kit and the 2100 bioanalyzer instrument (Agilent Technologies). The libraries were also quantified by quantitative polymerase chain reaction analysis using the Ion library quantification kit (Life Technologies) according to the manufacturer’s instructions. Amplified and clonal templates from libraries were generated by emulsion PCR using the Ion Proton Template OT2 kit. Sequencing was performed by multiplexing four barcoded templates on each Proton I chip and using the Ion Torrent Proton instrument (Life Technologies) according to the manufacturer’s instructions.

### Variant calling

The BAM files generated from deep sequencing and aligned by Ion Torrent Variant Server (ITV) had mean coverage depth of 1406; mean coverage of the 1 Mb target region was 95.6% at 100X (exceeding that in all but two samples) and 72.4% at 800X.

In order to perform tumor-normal analysis on the BAM files, we applied the following software packages (described in detail in Table 2), using blood or normal nasal samples as the paired-normal control: Ion Reporter (IR) [25], MuTect (MU) [26], and Varscan2 (VS) [27]. We also called variants in an unpaired manner using the Torrent Variant Caller (TVC) [28] and then constructed a straightforward tumor-normal variant subtraction routine by comparing the VCFs produced by TVC, a method we dubbed the “Poor Man’s somatic detector” (PM).

**Table 2:** Software versions used

Software	Label	Version	Notes
Ion Reporter	IR	IonReporter Version 0.1.2	IonReporter VCF employs TVC 4.2
Poorman’s	PM	Torrent Variant Caller 4.2	Subtract variants found in normal
Varscan	VS	varscan2.3.5	reject *p*-value *p*>10^−6^
MuTect	MU	muTect-1.1.7.jar	
MuTect-Gaps	MG	muTect-1.1.7.jar	nearby_gap_events added (see “Results” section)

Unmodified MuTect (MU) was not used in the analyses described because it produced so few variants

We used Variant Tools [29] to annotate the variants for each method with data from ANNOVAR [30], the 1000 Genomes Project (1 KG) [31] and the Exome Variant Server (EVS) [32]. For each method, we then excluded variants found in either 1KG or EVS. Finally, for the purposes of our final tabulation, we also excluded variants annotated as intronic or intergenic by ANNOVAR. An in-house analysis framework designed for quality control and reproducibility, called SyQADA (System for Quality Assured Data Analysis), was used to implement and execute all the bioinformatic pipelines described above.

#### Tuning VS and MU for ion torrent sequence data

The default settings for VS and MU were tuned for Illumina-generated sequence. We ran these programs on Ion Torrent data with those defaults and found that MU produced an order of magnitude fewer calls, and VS produced an order of magnitude more, than IR did. Furthermore, our visual inspection (discussed below) indicated poor performance of these two callers’ default settings on Ion Torrent data. We therefore explored modified options for these two programs, in order to find parameters that produced better performance.

For VS, we determined that excluding variants annotated with a *p*-value greater than 10^−6^ produced numbers of total variants more comparable to (but still larger than) the other callers. For MU, the default settings produced fewer than 10 total calls across all samples. Because MU had been designed to work with the GATK and Illumina data, we inspected the annotation file that is output alongside each MU-generated VCF to look for categories of exclusion criteria (flags in the annotation file) that were removing variants for reasons perhaps inappropriate for data from the Ion Torrent platform. We found two such candidate categories and created two new sets of MU VCFs, one containing those variants that were rejected only with the nearby_gap_events flag, and one for the clustered_read_position flag. Since we had already “validated” (by visual inspection) a complete set of variants at this point, we simply re-ran our caller concordance analysis including these new data. Variants with the annotation of clustered_read_position had very poor overlap with the existing set and further exhibited poor performance on the existing visually inspected variants, so we discarded these. However, MU output that included variants flagged as nearby_gap_events (“MG”) overlapped well with good performance on the existing visually inspected variants, so we randomly selected additional variants scored by MG (in the manner above) to provide the same basis for analysis as the other methods. After these new MG variants were visually inspected (although this new validation set was rich in MG variants, we chose to increase our sample from other callers at the same time, so this validation set was also anonymized with respect to the generating method), we re-ran the caller concordance analysis on the comprehensive set of variants. The numbers of variants and loci given in this text reflect this larger set. The script used to create the relaxed MG-generated VCF file is included in the Additional file 1.

### Visual inspection

To assess accuracies among the methods, we performed a rigorous visual inspection of all the variants found in common by two or more callers. For each method, we added to the inspection set a random selection of at most 50 variants called by that method only. For IR, there were only 23 such variants; VS alone called almost 309 variants that did not intersect with other callers). Thus, of the 637 loci identified in total, we examined 434 distinct genomic loci, intronic and exonic. Among these, 223 variants were obtained after filtering out those in intronic and intergenic regions. Several of the variants were identified at the same locus by different technologies in different patients, yielding multiple sample sets at a few loci.

#### Selection of variants for visual inspection

To derive a set of variants that we could use as a metric for quality of variant discovery, we used the Integrated Genome Viewer (IGV) [33] for visual inspection of a large fraction of the variants identified by the four packages.

To select the variants for inspection, we constructed an iPython notebook [34] employing Python and the Pandas data analysis framework [35] to identify common variants and produce our results tables.

#### Inspection workflow

We generated an anonymized list of variants for visual validation and created an IGV batch script to load the samples in which each variant was called, as well as the accompanying normal sample. Then four of us (AD, JF, HK, PS) each independently inspected between 10 and 50 variants and attempted to classify them into one of the following categories: *valid*, *bad*, or *homopolymer*, plus a temporary category *uncertain*. Together, we then reviewed our individual calls to reach consensus, and further categorized the uncertain sites into one of the three final categories. Then one of us (AD) inspected and classified all the remaining variants based on the common understanding gained in the “training set.”

Compared to other sequencing technologies (such as Sanger and Illumina), Ion Torrent sequencing output is relatively weaker around homopolymer runs (sequence regions of consecutive identical nucleotides) due to alignment slippage caused by the technology’s less accurate estimate of homopolymer length beyond a few bases [36]. In our PCR-based targeted sequencing, this was most conspicuous at the boundaries of amplicons, where homopolymers in the read caused cascades of variant calls at adjacent locations all through the read set; however, the problem was often present in the interior of amplicons as well.

**Fig. 1:**
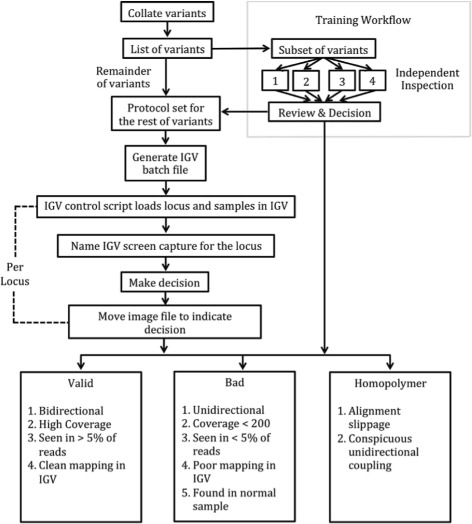
Variant Inspection Workflow. We used the depicted workflow to organize the variants called by the four software packages. We determined validity of variants via visual inspection after having established the categorization criteria described in the lower three boxes by consensus (Review and Decision, following independent inspections by individuals 1-4)

Our method for variant classification is described in Fig. 1. The criteria for variant classification are given below. IGV snapshots of examples are found in the Additional file 1, numbered to correspond to the criteria below:

A variant was called a *homopolymer* if the neighboring (within approximately 10 bp) reference sequence had a homopolymer run of at least 6 bases and the reads supporting the call showed

(i) obvious alignment slippage (seen as a locus where the called variant base is present on reference sequence in close proximity) [Additional file 1: Figure S1] or
(ii) conspicuously coupled calls at two or more nearby loci not detected in both forward and reverse reads [Additional file 1: Figure S2].

Rarely, an exception was made if the presence of a homopolymer run of 5 or 6 bases still produced good mappings and plausible variant calls, provided that the reference sequence surrounding it was sufficiently heterogeneous [Additional file 1: Figure S7]. A call that was not a homopolymer was classified simply as *bad* if it

(iii) did not have supporting reads on both strands [Additional file 1: Figure S3],
(iv) was found in less than 5% of the reads (with allele frequency precision supported by 200 reads) [Additional file 1: Figure S4],
(v) was found among reads that predominantly showed signs of poor mapping in IGV [Additional file 1: Figure S5], or
(vi) was found at more than 5% in the normal sample [Additional file 1: Figure S6] (in at least one case, PM called a false positive because a variant obviously present in comparable numbers in both tumor and normal was not called in the normal by TVC).

A typical *valid* call was bidirectional, had high coverage, was seen in more than 5% of the somatic reads but not found in the normal, and showed clean mapping in IGV.

For both our consensus calling and our complete classification of all putative variant sites, we built an IGV control script to refresh the IGV browser, move to the next location, load each sample exhibiting the call as well as a corresponding normal, sort the displayed reads by basecall, and then take an automatic snapshot (a PNG file) named for the locus. After evaluation, each snapshot was moved to the appropriate subdirectory (valid, homopolymer, bad). Some snapshots were tagged with descriptive terms to help in collective evaluation during consensus. This workflow blinded the inspector(s) to which method(s) called each variant, as well as to variant annotation and sequence context. The semi-automated workflow greatly reduced the amount of human input required to display and classify variants, consequently reducing the risk of clerical error (omission, duplication, misnaming, misclassification, etc.) during analysis of several hundred variants.

The validation set included variants found both in exons and introns, all of which underwent identical scrutiny. However, since our downstream focus is ultimately on biological interpretation of coding sequences, we subsequently restricted our analyses and summaries to exonic (non-intronic, non-intergenic) variants.

### Performance evaluation

We evaluated the performance of the methods by calculating sensitivity and positive predictive value (PPV). We chose positive predictive value because it is a useful measure in the diagnostics towards which this study is geared; furthermore, the domain of negative results necessary to calculate specificity is unknown. Each line in Table 3 reports sensitivity and PPV for an individual caller or a set of callers (a “strategy”). Sensitivity and PPV are formally defined in this context as follows: Let *V* be the number of variants that we classified as valid, let *v* be the number of valid variants correctly called by the strategy, and let *C* be the total number of variants called by the strategy. Then we define *PPV* to be *v*/*C*. Under the assumption that a somatic mutation existing in the DNA would have been detected by at least one of the four individual callers, *V* is a proxy for the true number of valid variants. From these quantities, we define *Sensitivity* to be *v*/*V*. We extrapolated the PPV for the individual callers and sensitivity for all strategies by assuming that the observed rates in the inspected variants also held in the uninspected variants to achieve an estimate of the true number of DNA mutations among *all* called variants.

**Table 3:** Performance of variant calling strategies

Strategy	Calls (C)	Valid (v)	PPV ^∗^	(SE, n)	Sensitivity ^∗^	(SE, *n*=223)
IR	127	64	0.50	(0.044, 127)	0.83	(0.025)
MG	106	41 ^∗^	0.39	(0.056, 76)	0.53	(0.033)
PM	199	71 ^∗^	0.36	(0.043, 124)	0.92	(0.018)
VS	381	26 ^∗^	0.07	(0.030, 72)	0.34	(0.031)
Any one	637	77 ^∗^	0.12	(0.022, 223)	1.00	(NA)
IR ∩ MG	42	40	0.95	(NA, 42)	0.52	(0.033)
IR ∩ PM	94	58	0.62	(0.050, 94)	0.75	(0.029)
IR ∩ VS	27	26	0.96	(NA, 27)	0.34	(0.031)
MG ∩ PM	40	39	0.97	(NA, 40)	0.51	(0.033)
MG ∩ VS	31	22	0.71	(0.082, 31)	0.29	(0.030)
PM ∩ VS	29	26	0.90	(NA, 29)	0.34	(0.031)
Any two	111	61	0.55	(0.047, 111)	0.79	(0.027)
IR ∩ MG ∩ PM	38	38	1.00	(NA, 38)	0.49	(0.033)
IR ∩ MG ∩ VS	22	22	1.00	(NA, 22)	0.29	(0.030)
IR ∩ PM ∩ VS	27	26	0.96	(NA, 27)	0.34	(0.031)
MG ∩ PM ∩ VS	22	22	1.00	(NA, 22)	0.29	(0.030)
Any three	43	42	0.98	(NA, 43)	0.55	(0.033)
IR ∩ MG ∩ PM ∩ VS	22	22	1.00	(NA, 22)	0.29	(0.030)
IR ∪ PM	232	77 ^∗^	0.33	(0.026, 157)	1.00	(NA)
(IR ∪ PM) ∩ (MG ∪ VS)	51	45	0.88	(0.046, 51)	0.58	(0.033)

Variant calls made and calls validated by visual inspection are given for each software package, as well as their intersections, excluding those variants identified by ANNOVAR as intronic or intergenic, which leaves 223 relevant SNPs collectively identified. The column labelled PPV shows Positive Predictive Value. Sensitivity and PPV were calculated as *Sensitivity*=*v*/*V* and *PPV*=*v*/*C*, respectively, where numerator *v* is the number of valid variants correctly called by a given caller or set of callers, denominator *V* is the extrapolated set of 77 valid variants (including 68 examined variants that we classified as valid plus 9 additional “valid” variants estimated via extrapolation), and denominator *C* is the total number variants called by all callers. The strategies described as “Any one,” “Any two,” and “Any three” each require the variant site to be identified by at least that many packages, which is effectively a union of sites identified by all the combinations in that section of the table. Asterisks (*) indicate values that were extrapolated. Standard error (SE) for PPV and Sensitivity is provided when *n*∗*min*(*p*,1−*p*)≥5, where *n* is the number of variants *examined* rather than the number *called*, because the latter is based on an extrapolation and does not contribute to estimated precision

### Validation by digital PCR

We studied select valid variants by digital poymerase chain reaction (PCR) using the QuantStudio platform (ThermoFisher Scientific). For digital PCR, 2 chips were run per sample per assay on the QuantStudio 3D AnalysisSuite software. Digital PCR assays were custom synthesis and run with a no-template and a negative control as part of assay validation; positive controls of these mutations were not available. Further details are in Additional file 1: Table S1.

## Results

We called variants with the four methods, Ion Reporter (IR), MuTect with Gaps (MG), Varscan2 (VS), and “Poor Man’s” (PM). Calling was done on somatic samples from 4 patients, 30 samples in all, using each patient’s corresponding normal sample as a contrast. The four methods together identified 1648 variants at 1318 genomic loci, of which 813 called variants at 637 loci were annotated as one of the following by ANNOVAR: exonic, UTR3, UTR5, stopgain, splicing or as non-coding RNA variants. We focused our analysis on these mutations.

**Fig. 2:**
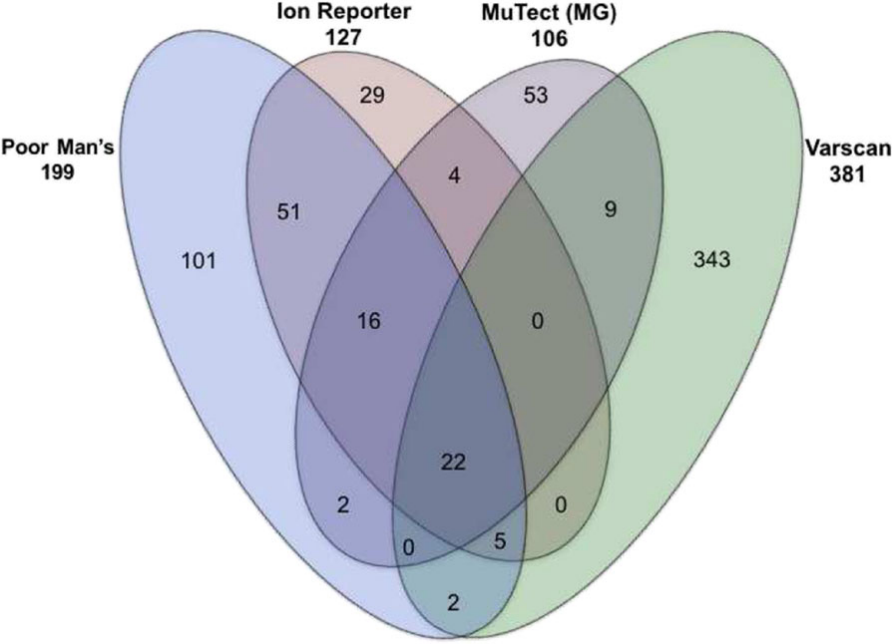
Venn Diagram of Number of Variants Grouped by Method Combinations. Listed under each method name are that method’s total called variants. (Note, areas are not scaled according to set size.)

The breakdown of the 637 called variant loci by method was as follows: 106 for MG, 127 for IR, 199 for PM, and 381 for VS. The overlaps among methods varied substantially by combination and are displayed in Fig. 2. The greatest agreement among methods was between IR and PM, perhaps not surprisingly, since both make use of Life Technologies’ TVC (Poor Man’s does tumor-normal subtraction of TVC’s VCF files); 74% of IR’s and 47% of PM’s variants were also identified by the other package. Over 70% of calls from IR were corroborated by at least one of the other callers, more than for any other method. Overall, this indicated a generally higher quality for IR calls.

A full description of results is in Table 3. In all, we examined a total of 223 putative variant sites after excluding those in intronic or intergenic regions. Of these, 68 were classified as valid. For the metrics in Table 3, homopolymer calls were lumped with other bad calls as “not valid”. Numbers marked with an asterisk(*) in the table were extrapolated from the PPV calculated on our 223 sites to estimate the number of additional valid variants that may exist in the sites we did not validate.

### Caller performance

Overall, among the individual packages, IR exhibited the best PPV,.50, with a corresponding sensitivity of.83. PM did achieve a higher sensitivity of.92, but its PPV was lower at.36. MG offered a modest increase in PPV over PM but had a much lower sensitivity. VS made the largest number of calls, the large majority of which were called by that program alone. Although VS identified approximately one-third of the estimated number of total valid calls, its PPV was low at.07. Notably, among 53 variants inspected visually that were called by either MG only or VS only, none were found to be valid.

### Aggregating results from multiple packages

To examine the value in aggregating results from multiple software packages, we considered the performance among called variants within all sets depicted in Fig. 2. Indeed, and as one might expect, corroboration of a variant call by an additional software package was a strong predictor of the call being classified as valid. Table 3 includes results for combinations of packages.

Perhaps the most obvious way to combine callers is to accept a variant if *any* method identified it as such. This strategy, labelled “Any One” in Table 3, had, by definition, a sensitivity of 1, but a PPV of only.12. To look at ways to increase PPV, we considered various combinations of callers. A more limited union of variants identified only by IR or PM (*IR*∪*PM*) retrieved *all* valid variants (sensitivity of 1) with a PPV of.33, a notable increase over the “Any One” strategy. Other strategies we examined included requiring a variant site to be agreed on by at least 2 or 3 of the packages, or even by all 4. We also evaluated specific combinations. The particular combination of IR *and* PM (*IR*∩*PM*) yielded the most favorable sensitivity among 2-method intersections (.75) with a corresponding PPV of.62, exceeding the PPV for IR alone(.50).

Two sets with MG (*IR*∩*MG* and *MG*∩*PM*) gave high PPVs, exceeding.95, with sensitivities above.50. Although selecting variants called by “Any Two” callers yielded a sensitivity of.79, the PPV of.55 was inferior to that of *IR*∩*PM*. If one can tolerate a sensitivity of.55, and generate calls from these four packages, the strategy of requiring *at least* 3 packages to identify a site yielded a PPV of.98. All 22 instances when all four packages called the same site were validated (*PPV*=1, sensitivity of.29). On these data, this result was actually inferior to using only *IR*∩*MG*∩*PM* (*PPV*=1, sensitivity of.49), which has the added virtue of not requiring the VS calculation. For the reader wishing to consider alternative strategies with these 4 variants callers and the unique sample set of multiple tumor samples from the same patient, we refer to a supplemental table (“Variant-Annotation-Dataframe.txt”), containing more complete read depth and allele frequency on a per-sample, per-variant level.

To provide a molecular (non-visual) form of validation to at least a few of the variant calls, we performed digital PCR. Variants were validated in two separate patients for two different genes, STK11 and KMT2D (each unique to one patient). For each variant, validation was observe in additional samples from the same patient. Further, in these examples, the allele frequencies from digital PCR strongly corresponded to those from NGS. Further details are available in Additional file 1: Table S1.

## Discussion

While our study included one type of tumor (NSCLC) from four patients, our goal here was to develop methods for better analysis of somatic mutations from IONT data. Although our results and conclusions are meant to be strictly interpreted for research rather than clinical settings, we surmise that the analytical methods we outline in our present study are generalizable to various somatic study designs, particularly using IONT sequencing data. Further, features of the NGS data or genome context could be incorporated to improve the performance of the individual callers, tuning some of the parameters of the algorithms. We did not attempt to do so in this study. Such strategies were explored elegantly in [37].

In our study we performed an evaluation of multiple strategies for identifying somatic point mutations in data from the Ion Torrent sequencing platform. We began with the existing software packages Ion Reporter (IR), Varscan (VS), and MuTect with a modification to include variants excluded only because of its “nearby_gap_events” filter (MG), which we considered a reasonable modification given the implicit presence of gaps in amplicon sequencing. We also implemented a simple “Poor Man’s” (PM) variant-subtraction routine based on the independent calls made by TVC on tissues and paired normal samples. We then applied various combinations of these callers, considering both unions and intersections of variant sets from individual methods to explore a range of tradeoffs among sensitivity and PPV. Since it is probable that some sites with a true variant were not picked up by any of the 4 methods, the estimates of sensitivity we give below serve as an upper bound.

Among the single callers, IR offered perhaps the most reasonable tradeoff of PPV and sensitivity, capturing 83% of all discovered variants (“sensitivity”) with a validation rate among them (“PPV”) of 50%. We also considered 2-way and higher order combinations to explore wider ranges of metrics that might be appropriate for different settings. For example, requiring a variant to be called by two methods increases PPV (over just one of the methods) at a corresponding cost of sensitivity. This may naturally be attractive when sorting through a large number of calls when high validity is important but visual inspection of reads is not practical. Combinations of IR, MG, and PM offered flexible ranges of accuracies. For example, if a 50% sensitivity is acceptable, then high PPV (95%) can be attained with 2-way combinations of MG and either IR or PM. However, if one had sufficient resources to perform some validation, or could accept some false positives, near-perfect sensitivity (all variants discovered) might perhaps be obtainable by running just two methods, IR and PM, and accepting initially anything called by either. Three- and four-way combinations yielded some examples of small increases in PPV and sensitivity over the 2-way callers IR ∪ MG and MG ∪ PM, but given our limited sample size it is unclear how quantifiable this is.

Where specificity approaches unity, as we observed with “Any Three” callers and for particular 2-way combinations, the value of visual validation becomes debatable. It may then be necessary only to validate the larger fraction that are called by no more than 2 callers. Additional filters for homopolymers, low-coverage calls, and higher standards for presence in normal, could also easily be applied to the results. This might improve calling enough to obviate the need for running one or more of the methods.

In particular, running VS may offer many useful results or features but, for practical purposes in these data, should be run in combination with another caller, as we have done in this evaluation, particularly with IR or a version of PM. However, in these data, VS is largely redundant with MG, and, since none of the 34 examined variants called by VS only was found to be valid, one might consider eliminating VS from the caller set without much adverse effect. The advantage of retaining VS is in refining the PPV of other callers, at the expense of roughly doubling the computation requirement.

Not surprisingly, accuracy was relatively better in exonic regions than in introns. This matches intuition because the exons are typically more complex regions than introns, meaning that there are likely to be fewer homopolymer regions in the exome than in intronic regions of the genome (though of course, runs of homopolymeric codons do occur). Since our data set is specifically targeted to a gene set (Ion Torrent’s Comprehensive Cancer Panel, 409 genes), we intend to incorporate exclusion of intronic calls into our prospective workflow.

We noted variants that were found in all or the majority of sample CNBs from a particular patient. Some variants exhibit more intra-tumoral heterogeneity (ITH) and were found in few CNBs or in one biopsy, albeit these were relatively rarer. It cannot be neglected that, using the select sequencing platform and calling strategies, variants are more challenging to detect in more complex ITH. Nonetheless, the presence of variants in CNBs of one patient but not in biopsies from another patient provides, indirectly, confidence on the validity of these variants. Previous large-scale sequencing efforts have underscored driver mutated genes in NSCLC [6–8, 38, 39]. In our set of four patients, we found “valid” variants in a couple of mutated drivers such as *KEAP1*, *STK11*, *KMT2D* (also known as *MLL2*) and *TP53*. It is important to mention that these mutated driver genes were detected in specific patients and in the majority of tumor samples from those specific patients. Also, one patient (smoker lung adenocarcinoma) exhibited both driver *TP53* and *KEAP1* mutations, a mutation profile that has been previously described to be typical of smoker lung adenocarcinoma patients [7]. Based on these initial observations (identification of mutated bona fide NSCLC drivers), we draw at least some confidence in our variant calling results. Nonetheless, using digital PCR (QuantStudio Life Technologies platform), we confirmed the presence of variants in *STK11* and *KMT2D* in two different patients (Additional file 1: Table S1). Because we performed a limited validation analysis on a number of variants, the possibility of failed validation (either due to false positives by the calling algorithms or false negatives due to limitations of using custom primers and digital PCR) cannot be ruled out.

It is important to note that our study utilized frozen NSCLC tissues that typically display adequate quality of double-stranded genomic DNA and not FFPE processed specimens that are typically used for NGS assays in the clinic and comprise artificial base changes due to fixation. Although FFPE specimens better emulate the expected quality of sequencing in the clinic, we opted to use frozen tissues here, since this study represents our first attempt to compare and contrast different calling algorithms to improve base calling quality from IONT sequencing data. It is important to mention that the Ion Torrent sequencing platform has several limitations that we do not probe in the present study. Identification with precision of small insertions and deletions (indels) using the Ion Torrent platform is challenging. This limitation is significant in studies of NSCLC since actionable (responsive to tyrosine kinase inhibitors) exon 19 deletions in the *EGFR* oncogene are common in non-smoker NSCLC patients (mainly of the adenocarcinoma subtype). In this context, the necessity for caution arises in interpreting sequencing results, particularly in the clinical (e.g. CLIA) setting, generated by the Ion Torrent platform. A more complete evaluation of variants in a study like ours would ideally include additional automated validation procedures, such as orthogonal sequencing technology, beyond simply evaluating variants by visual inspection. To partly address this need, we did validate a couple of the discovered variants by digital PCR, variants in STK11 and KMT2D. Yet, these aforementioned limitations notwithstanding, our study’s findings warrant further investigation of the outlined analytical methods in future studies aimed at confidently distinguishing mutations from sequencing artifacts in FFPE specimens sequenced with IONT.

## Conclusion

This investigation provides a rough set of parameters with which an investigator can design a variant calling, and accompanying validation, strategy for a set of experiments. It also suggests improvements in variant calling for such data based on tiered strategies (such as accepting any variant called by 3 methods and visually inspecting those called by fewer). Of the several strategies explored here, selection of the one to be considered optimal may be dependent on the tissues being investigated, the expected tumor cellularities (and thus cancer cell fractions), and the resources available for validation (visual or molecular), as well as other issues intrinsic to an individual study design.

## Additional file

Additional file 1 Supplementary Materials. (PDF 1208 kb)

## Abbreviations

CNB: Core needle biopsy
FFPE: Formalin-fixed paraffin embedded
IGV: Integrated genome viewer
IONT: Ion-torrent
IR: Ion reporter
MG: MuTect with nearby gap events
MU: MuTect
NGS: Next generation sequencing
NSCLC: Non-small cell lung cancer
PM: Poor man’s somatic detector
PPV: Positive predictive value
SNVs: Single nucleotide variants
SyQADA: System for quality assured data analysis
TVC: Torrent variant caller
VS: VarScan2
