# Supplementary material for: Strategies for identification of somatic variants using the Ion Torrent deep targeted sequencing platform
Source: BMC Bioinformatics. 2018 Jan 4;19:5. doi: 10.1186/s12859-017-1991-3 (PMC5753459; doi:10.1186/s12859-017-1991-3)

Supplementary Materials

Mutation	S1 (T)		S2 (CNB4)		S3 (CNB6)		S4 (CNB8)		Normal	
STK11	NGS	dPCR	NGS	dPCR	NGS	dPCR	NGS	dPCR	NGS	dPCR
662CT	.52 , 21	.67	.47 , 47	.45	.39 , 66	.51	.39 , 423	.46	n/a	.05
	S1 (T)		S2 (CNB1)		S3 (CNB3)				Blood	
KMT2D	NGS	dPCR	NGS	dPCR	NGS	dPCR			NGS	dPCR
5766GC	.30 , 3954	.27	.09 , 1066	.10	.20 , 1048	.22			n/a	0

Table S1: The STK11 and KMT2D variants were detected (by Ion Torrent sequencing) and validated (by digital PCR) in separate patients. NGS entries are given as `allele frequency , read depth` based on output from Ion Reporter. The Normal and Blood samples were used as comparators during analysis. For digital PCG (dPCR), 2 chips were run per sample per assay on the QuantStudio 3D AnalysisSuite software. Digital PCR assays were custom synthesis and run with a no-template and a negative control as part of assay validation; positive controls of these mutations were not available. Two additional assays with custom primers were performed; these did not validate mutation calls. It is possible that additional tuning or optimization is needed for these custom primers.

MuTect Stringency Relaxation Filter

The accompanying text file, `relax_mutect_filter.py.txt`, illustrates the filter used to relax the stringency of MuTect to include those variants rejected for `nearby_gap_events` only.

Examples of Visually Inspected False Positive Variant Calls

The following figures display example snapshots of Integrated Genome Viewer (IGV) [33] showing 5 classes of called variants that we deemed to be false positives upon visual inspection. Each figure represents the alignment of reads 40 bases on either side of a called variant at the locus indicated by the column at the center of the image. Displayed reads are a random sample of the reads covering the locus, and are grouped by sample and ordered from top to bottom by variant (the interactive IGV interface also provides detailed information on the exact counts of alternative base calls and read directions in an information box that appears upon placing the mouse over the locus). A sampling of the variant calls is seen at the top of the locus column for each sample. The row at the bottom of the image shows the reference DNA sequence of the 81-base context.

Figure S1: *False-positive variant call: (i) obvious alignment slippage (seen as a locus where the called variant base is present on reference sequence in close proximity).*

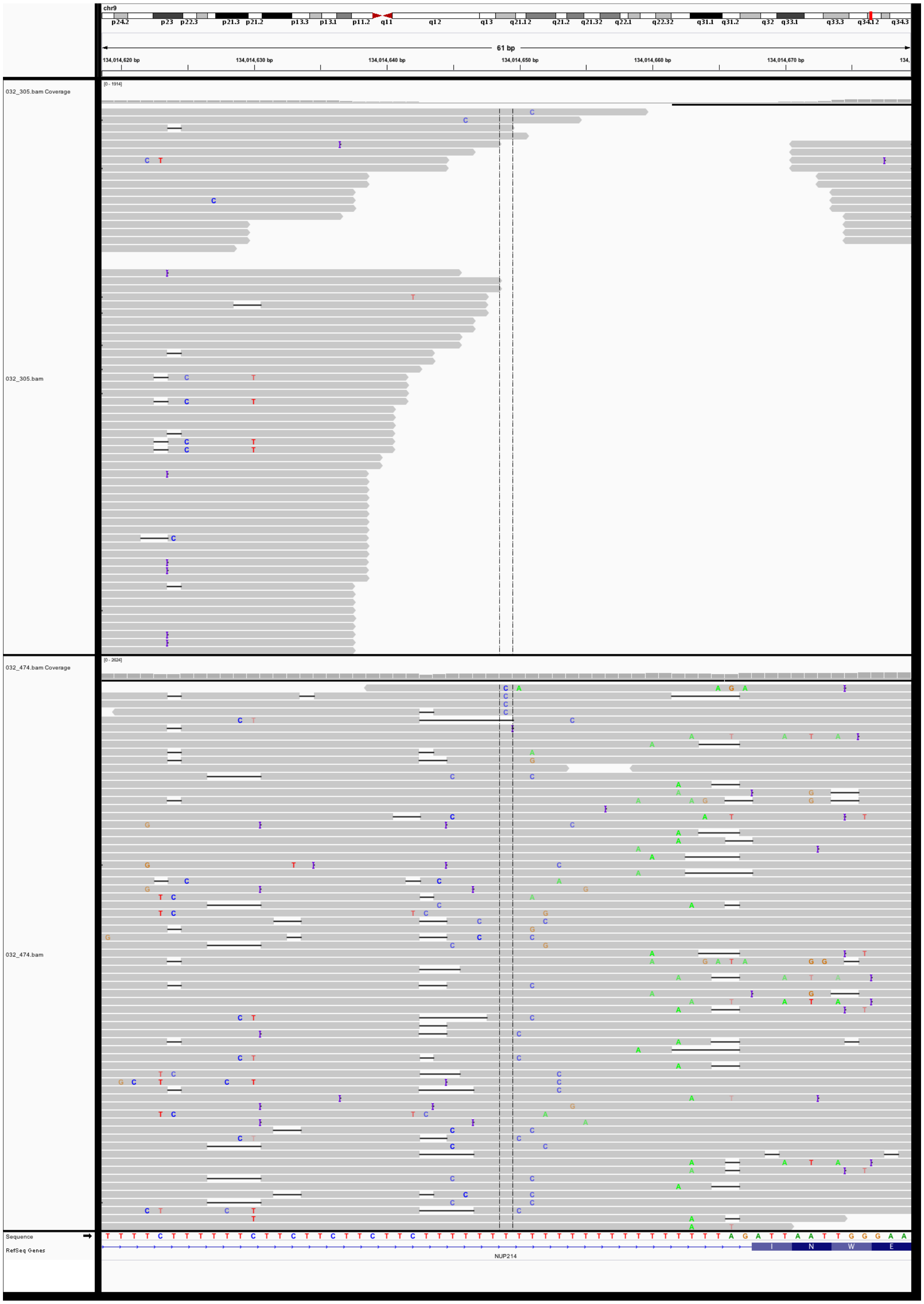

Figure S2: *False-positive variant call: (ii) conspicuously coupled calls at two or more nearby loci not detected in both forward and reverse reads.*

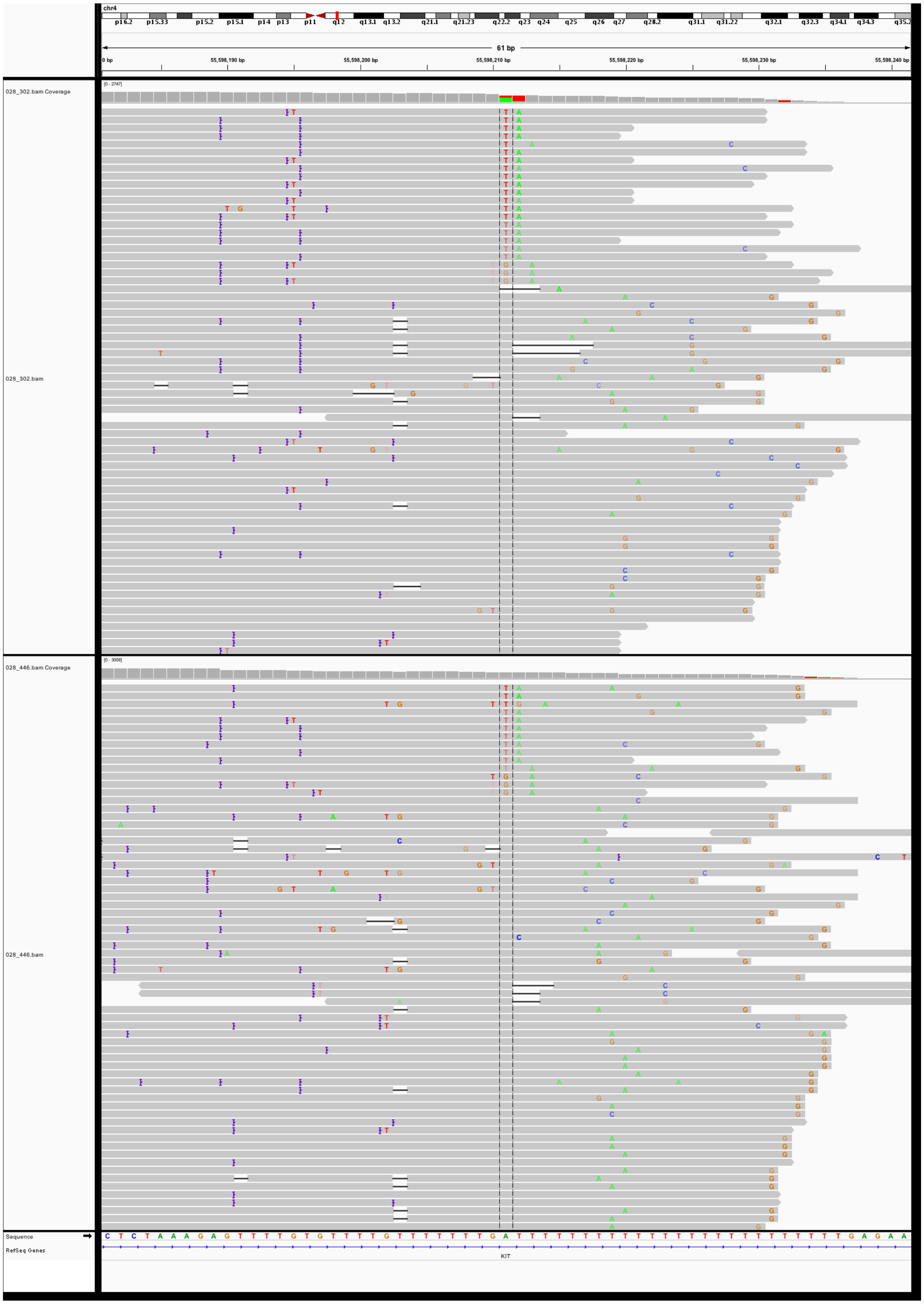

Figure S3: *False-positive variant call: (iii) did not have supporting reads on both strands.*

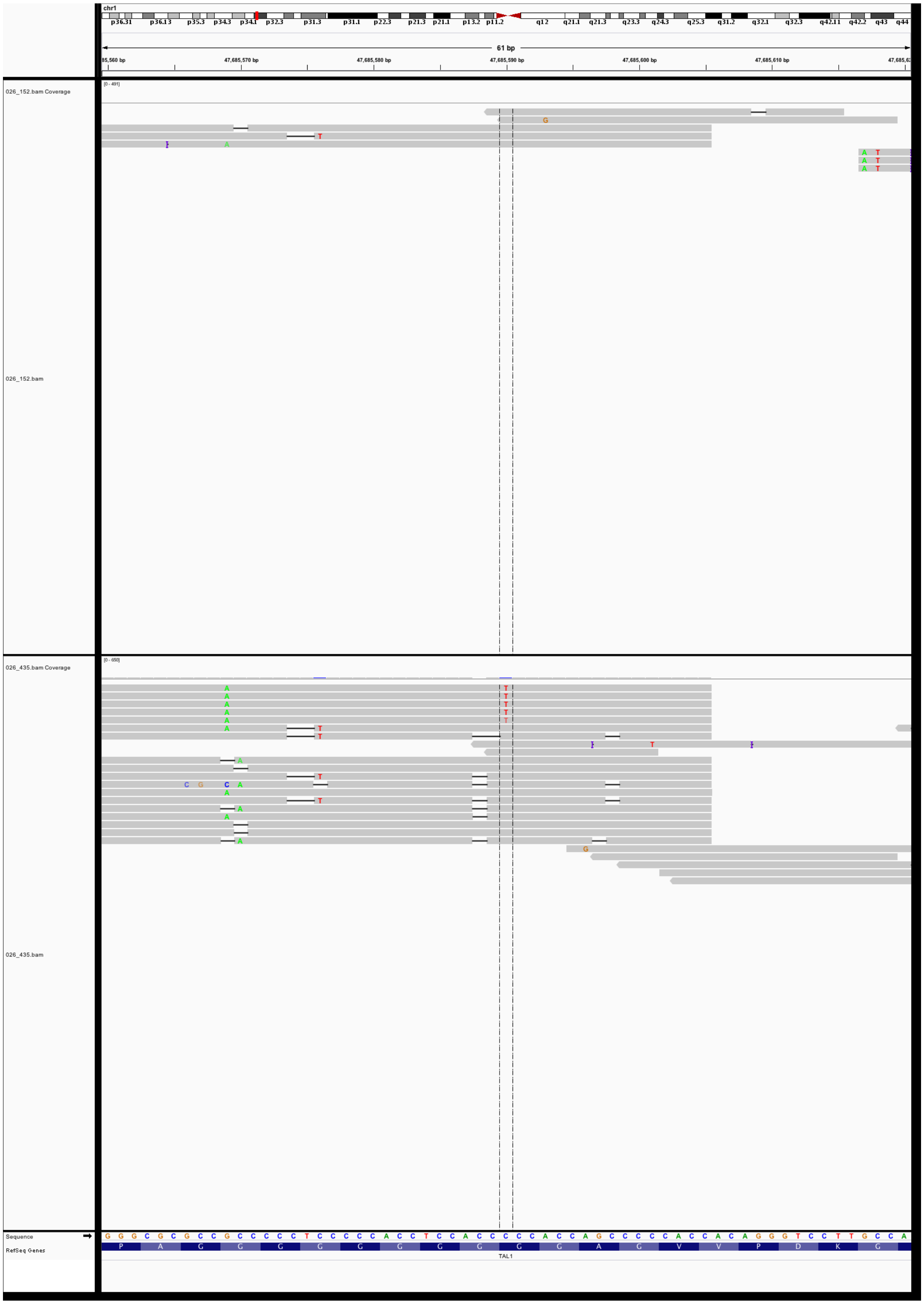

Figure S4: *False-positive variant call: (iv) was found in less than 5% of the reads (with at least 200 reads.*

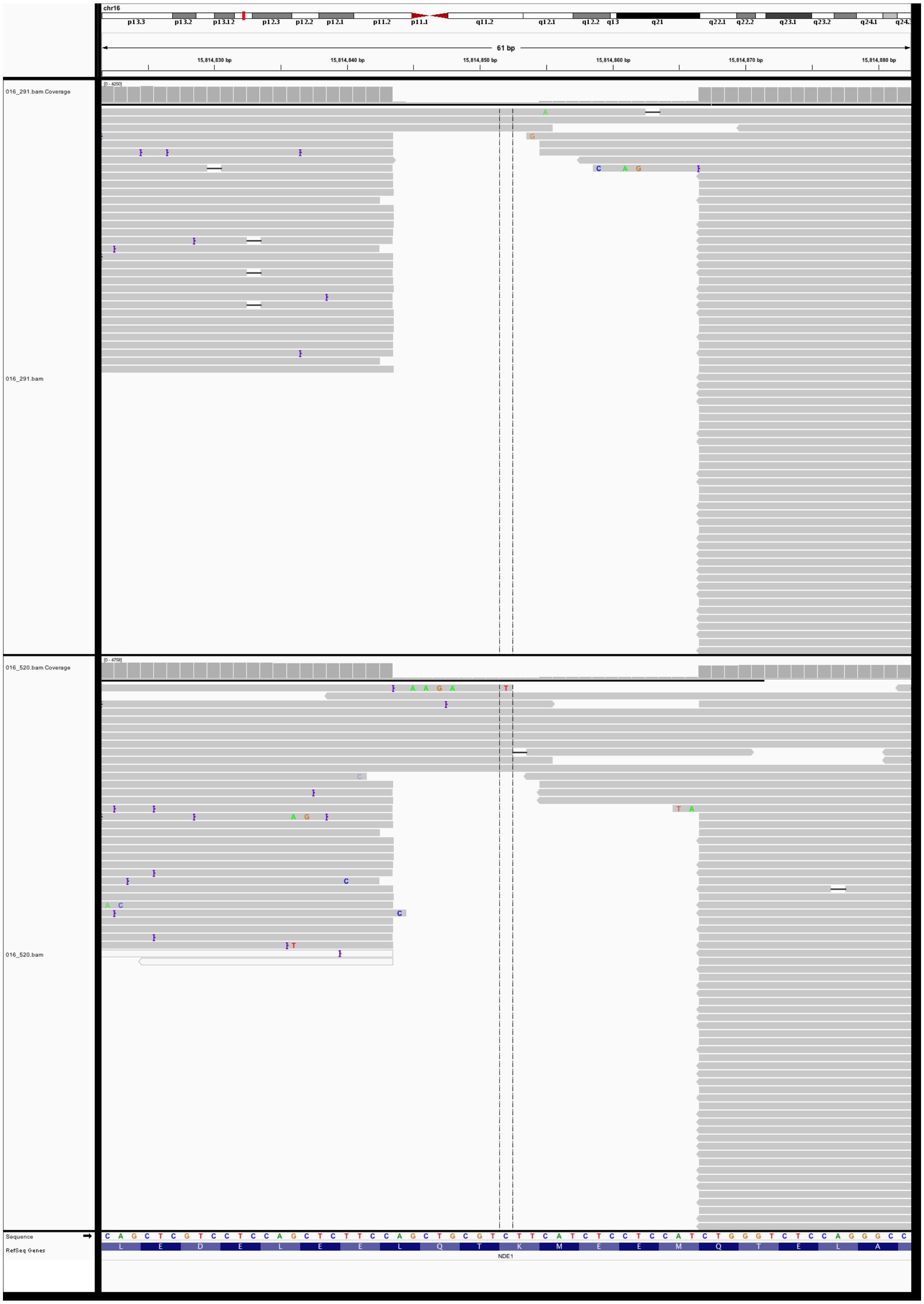

Figure S5: *False-positive variant call: (v) was found among reads that predominantly showed poor mapping in IGV.*

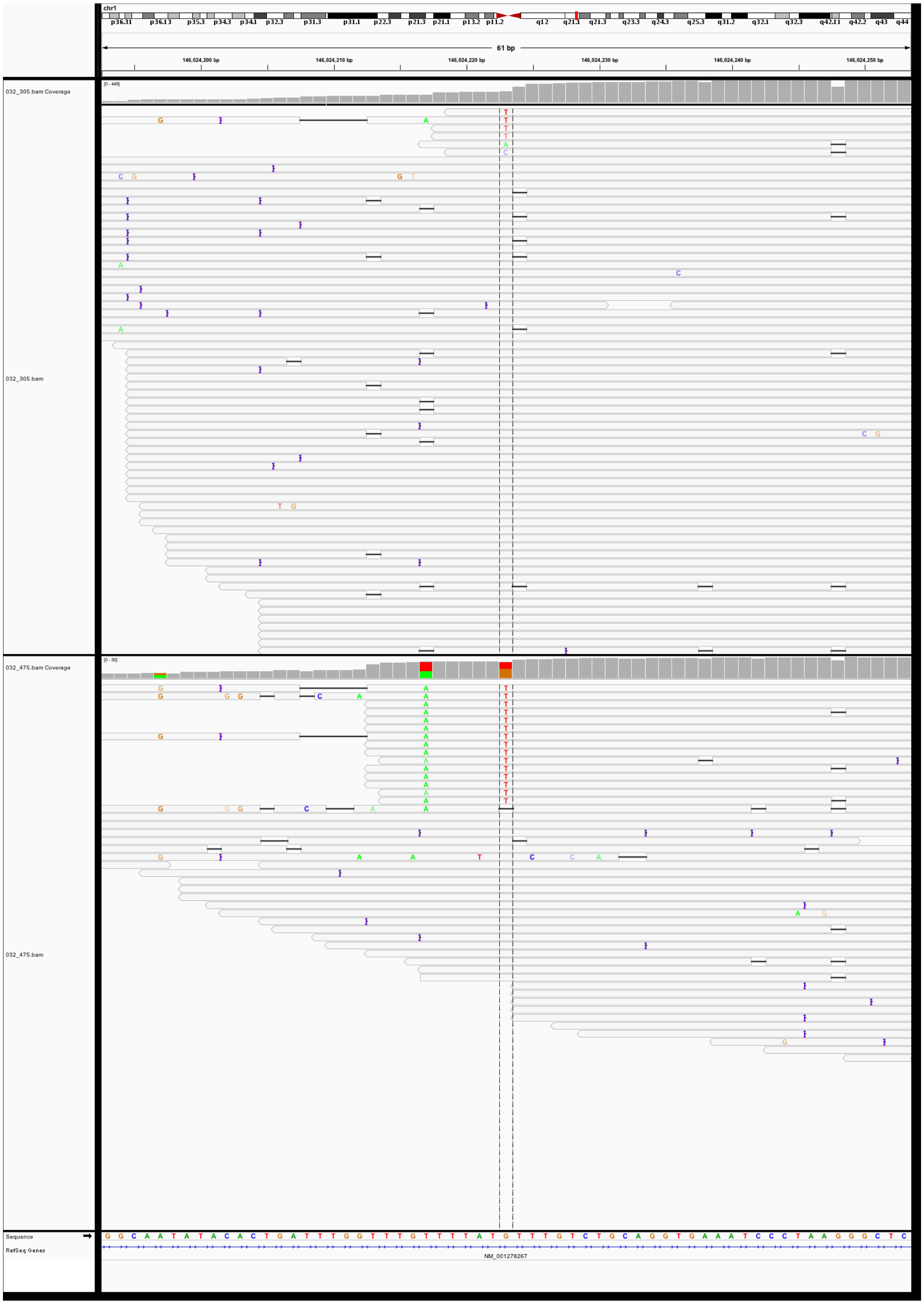

Figure S6: *False-positive variant call: (vi) was found at more than 5% in the normal sample.*

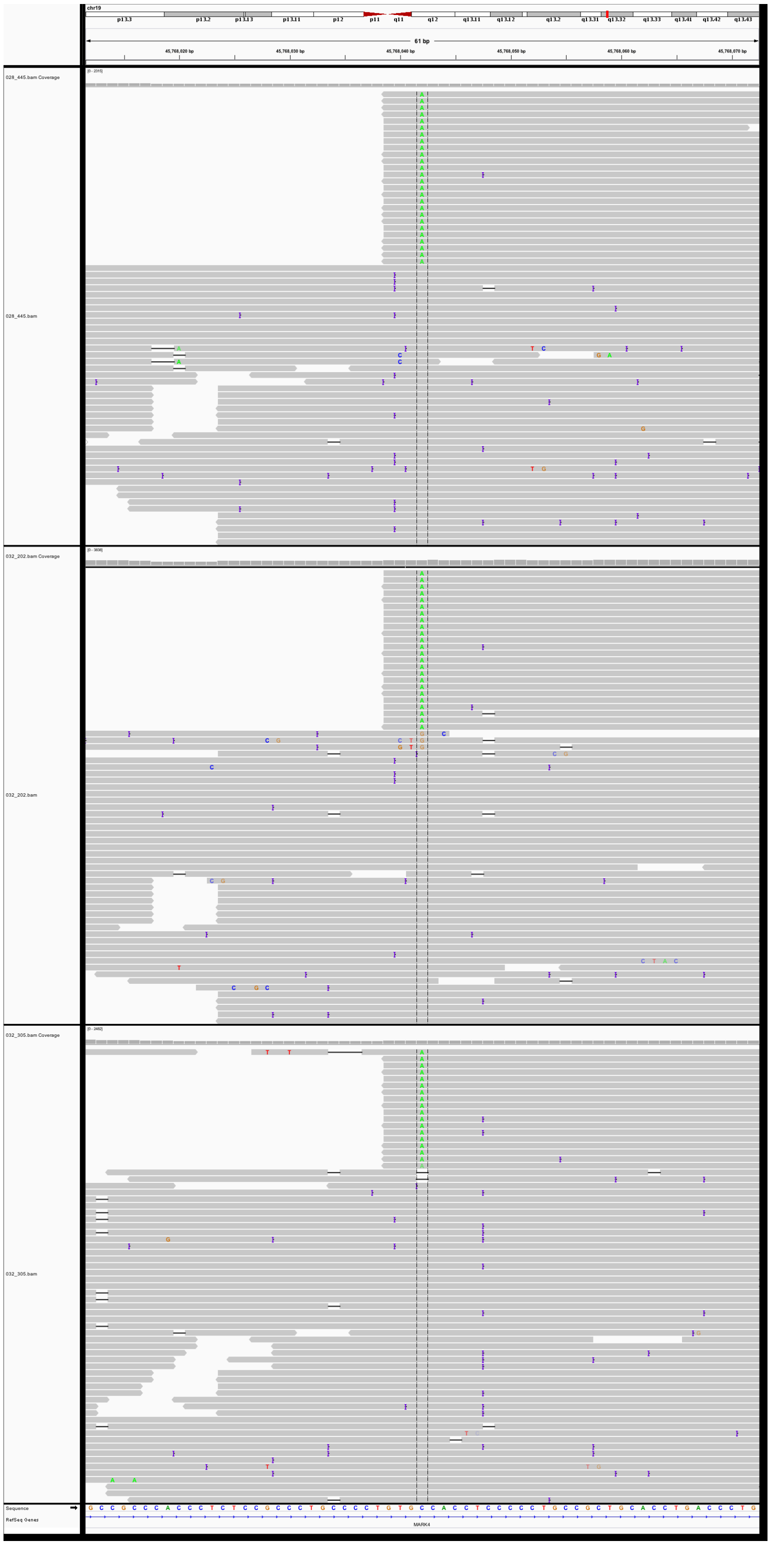

Figure S7: *True-positive exception: a homopolymer run of 5 or 6 bases still produced good mappings and plausible variant calls, provided that the reference sequence surrounding it was sufficiently heterogeneous*

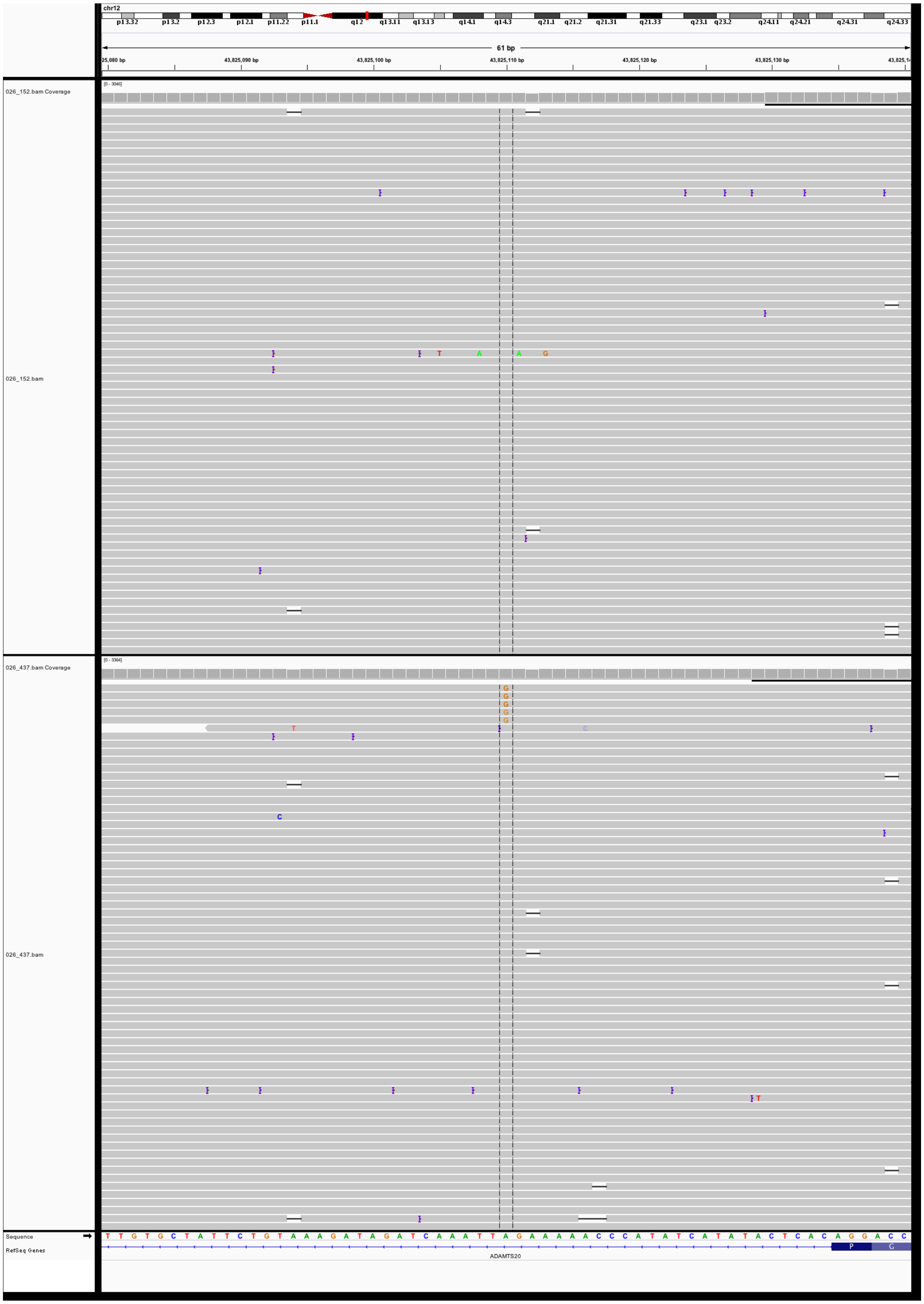

Supplement: Supplementary file 1 — Supplementary Materials. (PDF 1208 kb) [file 12859_2017_1991_MOESM1_ESM.pdf]
